# Introduction of a 6-cyano group in 14-oxygenated *N*-methylmorphinans influences *in vitro* and *in vivo* pharmacological activities

**DOI:** 10.1186/1471-2210-11-S2-A26

**Published:** 2011-09-05

**Authors:** Valeria Follia, Mario D Aceto, Louis S Harris, Andrew Coop, Helmut Schmidhammer, Mariana Spetea

**Affiliations:** 1 Department of Pharmaceutical Chemistry, Institute of Pharmacy and Center for Molecular Biosciences, University of Innsbruck, 6020 Innsbruck, Austria; 2 Department of Pharmacology and Toxicology, Medical College of Virginia, Virginia Commonwealth University, Richmond, VA 23298-0613, USA; 3 Department of Pharmaceutical Sciences, University of Maryland, School of Pharmacy, Baltimore, MD 21201, USA

## Background

Being a disabling symptom of many medical conditions, effective pain control is one of the most important therapeutic priorities. Morphine and other opioid drugs produce analgesia primarily through μ opioid (MOP) receptors, which mediate beneficial but also the non-beneficial actions. Appropriate identification of novel opioid analgesics may reduce complications and improve patient compliance. It was reported that hydrazones, oximes, carbazones and semicarbazone derivatives of morphinan-6-ones, e.g. dihydromorphinone or oxymorphone, exhibit high affinity at the MOP receptor [1]. Since most of these structures show high antinociceptive potency while having less pronounced side effects, it remains a promising task to convert the carbonyl group of morphinan-6-ones into various functionalities. In this study, we aimed to investigate the effect of the replacement of the 6-keto function with a 6-cyano group on *in vitro* and *in vivo* pharmacological profiles.

## Methods

Binding affinities at opioid receptors were determined using competition binding assays in rodent brain membranes. *In vitro* [^35^S]GTPγS functional assays were performed with Chinese hamster ovary (CHO) cell membranes expressing human opioid receptors. Antinociceptive activities were assessed in mice using tail-flick, hot-plate and writhing tests.

## Results

Replacement of the 6-keto group by a 6-cyano substituent in *N*-methylmorphinan-6-ones leads to qualitative and quantitative differences in the interaction with opioid receptors. Consequently, we have conducted a comparison of the biological activities of the 6-cyanomorphinans to those of structurally-related opioids, oxycodone, oxymorphone and of the clinically relevant morphine. The 6-cyanomorphinans displayed high affinity and behaved as agonists at the MOP receptor. When tested *in vivo*, they acted as potent antinociceptive agents after subcutaneous administration, being more active than the 6-keto analogues. The presence of a 14-methoxy or a 14-cinnamyloxy group instead of a hydroxy group not only increased *in vitro* opioid activity at the MOP receptor, but also enhanced the antinociceptive potency.

## Conclusions

Our findings revealed that targeting position 6 in the morphinan skeleton represents a viable approach for tuning the pharmacological properties of this class of opioids. Appropriate molecular manipulations could afford ligands that, besides their scientific value as pharmacological tools, may also have the potential of emerging as novel analgesics with fewer side effects compared to currently available treatments.
